# Structure and Function Relationship of the Autotransport and Proteolytic Activity of EspP from Shiga Toxin-Producing *Escherichia coli*

**DOI:** 10.1371/journal.pone.0006100

**Published:** 2009-07-01

**Authors:** Jens Brockmeyer, Sabrina Spelten, Thorsten Kuczius, Martina Bielaszewska, Helge Karch

**Affiliations:** 1 Institute for Hygiene and the National Consulting Laboratory on Hemolytic Uremic Syndrome, University of Münster, Münster, Germany; 2 Department of Medicine B, University Hospital Münster, Münster, Germany; University of Würzburg, Germany

## Abstract

**Background:**

The serine protease autotransporter EspP is a proposed virulence factor of Shiga toxin-producing *Escherichia coli* (STEC). We recently distinguished four EspP subtypes (EspPα, EspPβ, EspPγ, and EspPδ), which display large differences in transport and proteolytic activities and differ widely concerning their distribution within the STEC population. The mechanisms underlying these functional variations in EspP subtypes are, however, unknown.

**Methodology/Principal Findings:**

The structural basis of proteolytic and autotransport activity was investigated using transposon-based linker scanning mutagenesis, site-directed mutagenesis and structure-function analysis derived from homology modelling of the EspP passenger domain. Transposon mutagenesis of the passenger domain inactivated autotransport when pentapeptide linker insertions occurred in regions essential for overall correct folding or in a loop protruding from the β-helical core. Loss of proteolytic function was limited to mutations in Domain 1 in the N-terminal third of the EspP passenger. Site-directed mutagenesis demonstrated that His^127^, Asp^156^ and Ser^263^ in Domain 1 form the catalytic triad of EspP.

**Conclusions/Significance:**

Our data indicate that in EspP i) the correct formation of the tertiary structure of the passenger domain is essential for efficient autotransport, and ii) an elastase-like serine protease domain in the N-terminal Domain 1 is responsible for the proteolytic phenotype. Lack of stabilizing interactions of Domain 1 with the core structure of the passenger domain ablates proteolytic activity in subtypes EspPβ and EspPδ.

## Introduction

Gram-negative bacteria have developed various pathways to secrete proteins into their milieu. Among the mechanisms characterised, the autotransporter, or Type V secretion pathway is apparently the simplest. The N-terminal signal peptide is required for the recognition by the sec machinery which mediates the transport through the inner membrane, whereas the β-domain at the C-terminus of the autotransporter inserts into the outer membrane and facilitates the secretion of the transported passenger domain to the extracellular milieu [1]. More than 800 different autotransporters are known, forming the largest group of secreted proteins in Gram-negative bacteria [2], [3].

The serine protease autotransporters of *Enterobacteriaceae* (SPATE) constitute a subfamily of autotransporters that secrete passenger domains displaying serine protease activity. SPATE proteins have, with some exceptions [4], only been identified in pathogens and are among the predominant proteins secreted by these organisms [5], underlining their potential role in pathogenesis. Though sharing similar structural features, the SPATE proteins appear to be functionally diverse [6].

EspP (extracellular serine protease, plasmid-encoded) is a member of the SPATE family encoded on the large virulence plasmids of Shiga toxin (Stx)-producing *Escherichia coli* (STEC) [7]. STEC are emerging pathogens worldwide and, like the best known member of this group, *E. coli* O157:H7, cause a spectrum of diseases ranging from uncomplicated diarrhoea to haemorrhagic colitis (HC) and the life-threatening haemolytic uraemic syndrome (HUS) [8], [9]. Although Stxs are considered cardinal virulence traits of this group of organisms, additional virulence factors contribute to the pathogenesis of STEC infections [8], [10]–[12]. One such factor is EspP, one of the most abundant proteins in culture supernatants of STEC strains [5, Brockmeyer, unpublished observation].

EspP cleaves coagulation factor V in human plasma [6], [7], and has been used as prototype to analyse different aspects of autotransportation in recent years. This work demonstrates the complexity of the mechanistic details of this secretion pathway [13]–[15].

We have recently studied the distribution, biological activity and structural aspects of EspP in a large collection of STEC clinical isolates and distinguished four subtypes of EspP (α, β, γ and δ). These isoforms differ substantially in their structure and functions [16]. EspPα (produced mainly by serotypes associated with severe disease including HUS [17]) and EspPγ are highly proteolytic and efficiently autotransported. EspPβ and EspPδ were either not secreted or were proteolytically inactive. These findings provide an opportunity to determine the critical modifications in the EspP subtypes that contribute to these phenotypes. We have therefore conducted transposon-based linker permissive mutagenesis and site-directed mutagenesis to map regions crucial for transport or proteolytic activity throughout EspP. Homology modelling of the EspP passenger was applied for the structure-based analysis of respective mutants to gain a deeper understanding of the molecular mechanisms underlying proteolytic and autotransport activity in general and loss of function in EspP subtypes in particular.

## Results

### Generation of EspP transposon mutants

**Figure 1:**
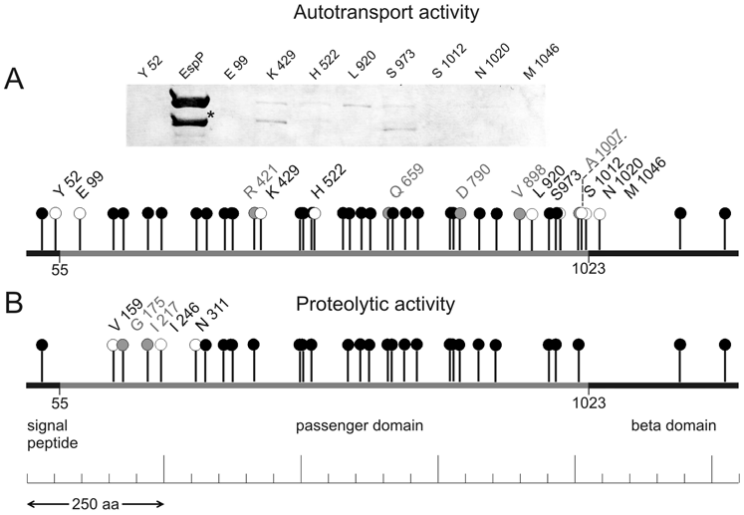
Representation of EspP linker mutants inserts and phenotypes. A. Insert position and transport activity of linker mutants. Immunoblot of concentrated culture supernatants of wild-type EspP (strain DH5α/pB9-5) and secretion-incompetent linker mutants where only trace amounts or no EspP was detectable. The EspP autodegradation band is indicated by asterisk. Insert positions of single mutants within EspP holoprotein are symbolized by pins, transport activity is indicated by colour code (black: transport activity comparable to wild-type, grey: residual activity (∼10–20%), white: loss of transport activity). Mutants displaying loss of function are indicated by construct names. B. Linker mutants assayed for proteolytic activity. Activity of single mutants is illustrated by colour code (see A for description), and construct names of inactive mutants are indicated.

To analyse structure-function relationships, EspP clone DH5α/pB9-5 [7] was subjected to permissive linker transposon mutagenesis to generate mutants harbouring in-frame 15 bp insertions at different positions of *espP*. We obtained 45 transposon mutants with in-frame insertion in the open reading frame of *espP*, resulting in a pentapeptide linker of the corresponding mutant protein. The linker insertions were distributed almost randomly over the holoprotein, but with a diminished number of mutants within the β-domain, as evidenced by nucleotide sequencing (Table 1 and Fig. 1).

**Table 1:** Phenotypes and insert positions of EspP linker mutants.

Insert position in mutant	Secretion competence	Proteolytic activity
K37	•	•
Y52	○	NA
E99	○	NA
V159a	•	○
V159b	•	○
G175	•	□
I217	•	□
I246	•	○
N311	•	○
N329	•	•
D362a	•	•
D362b	•	•
G380	•	•
R421	□	•
K429	○	NA
V500	•	•
T504	•	•
N519	•	•
H522	○	NA
Y575	•	•
M588	•	•
N613	•	•
T631	•	•
Q659	□	•
D669	•	•
N690	•	•
T709	•	•
N772	•	•
Y779a	•	•
Y779b	•	•
D790	□	•
K832	•	•
T858	•	•
V898	□	•
L920	○	NA
K956	•	•
Q968	•	•
S973	○	NA
A1007	□	•
S1012	○	NA
N1020	○	NA
M1046	○	NA
L1192	•	•
N1271a	•	•
N1271b	•	•

Constructs are listed in order from N- to C-terminus of the EspP holoprotein. Nomenclature of mutants is according to the insert position of the 5 amino acid linker in the primary sequence of EspP. Mutants designated “a” and “b” represent independent insertions at identical amino acid positions. The resulting phenotype is illustrated as follows: • active, ○ inactive, □ residual activity of ∼10–20%. NA, not applicable because of absent secretion competence.

### Effect of linker insertions on secretion efficiency

Fourteen of the 45 transposon mutant constructs displayed severely reduced (80%–100%) secretion as evidenced by protein immunoblot analysis of culture supernatants. The insert positions in the secretion-deficient constructs were not distributed evenly over EspP but accumulated in three distinct parts of the protein. Eight of 14 secretion-deficient constructs sustained inserts in the C-terminal part of the passenger domain or the N-terminus of the β-domain (Fig. 1A). In two constructs without transport activity, inserts were localised to the interfacing region separating signal peptide and the N-terminal passenger domain. In the remaining constructs, the linker was located in the central part of the passenger domain (from amino acid 420 to 660) (Fig. 1A). In accordance with the findings of Brunder and colleagues [7] we were not able to detect intracellular amounts of EspP in DH5α/pB9-5 suggesting that expression of the protein is followed by rapid secretion. In contrast, the secretion-incompetent linker mutants Y52, K429, H522, L920 and S1012 displayed detectable intracellular amounts of the passenger domain of EspP indicating accumulation of the protein in the late steps of biogenesis (data not shown).

### Proteolytic activity of EspP transposon mutants

The effects of linker insertions on proteolysis were determined in the 36 transposon mutants where EspP was found in culture supernatants (Table 1). Loss of proteolytic activity was restricted to constructs with insertions in the N-terminal third of the passenger domain. All mutants harbouring their inserts between Val^159^ and Asn^311^ displayed reduced proteolytic activity against the well characterised EspP substrates porcine pepsin A [7] and the chromogenic oligopeptide Ala-Ala-Pro-Leu-pNA [6]. Constructs G175 and I217 retained residual activities of ∼20%, but mutants V159, I246 and N311 lacked all proteolytic activity (Fig. 1B), indicating that the structural basis for proteolytic activity is encoded solely within this region.

Taking into account that the proteolytic activity of serine proteases is, in general, mediated by catalytic triads consisting of aspartic acid, histidine and serine residues [18], [19] we sought to elucidate the position of the catalytic triad in the EspP passenger domain. Fink and colleagues previously identified the catalytic triad in the Hap autotransporter of *Hemophilus influenzae* and demonstrated that this motif is conserved among related autotransporters displaying serine protease activity [20]. We aligned therefore the N-terminal third of the passenger domain of EspP with Hap to determine sequence conservation within this region using the AlignX tool in Vector NTI software package (Invitrogen Inc., Karlsruhe, Germany) (data not shown). This alignment demonstrated identical amino acid composition of Hap and EspP at positions forming the active centre of Hap (His^98^, Asp^140^ and Ser^243^), indicating, that the catalytic triad of EspP might be encoded at the respective positions (His^127^, Asp^156^ and Ser^263^) of the EspP passenger domain. To pursue these findings experimentally, we conducted site-directed mutagenesis of the corresponding residues to alanine and assessed the proteolytic activity of the resulting EspP mutants. All three constructs (H127A, D156A and S263A, respectively) completely lacked proteolytic activity, further substantiating that His^127^, Asp^156^ and Ser^263^ form the active centre of the EspP passenger domain.

Interestingly, the nonproteolytic mutant N311 contains the linker insertion next to point mutations Y313F and N316K, which we identified previously in all proteolytically inactive samples of EspPβ and EspPδ [16]. We performed site-directed mutagenesis to determine if these mutations underlie the proteolytic inactivity in these EspP subtypes. The double mutant Y313F/N316K showed severely reduced (∼45%) proteolytic activity when compared to wild- type EspP from DH5α/pB9-5, underlining that these alterations contribute significantly to the loss of proteolytic activity in EspPβ and EspPδ.

### Homology modelling of the EspP passenger domain

**Figure 2:**
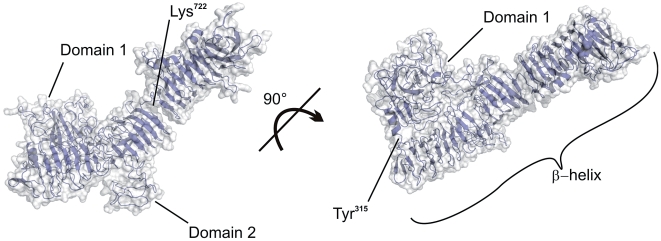
EspP homology model. Ribbon diagram (blue) with overlaid surface displaying the overall structure of the homology model of the EspP passenger. Domains 1 and 2 as well as the β-helical stem are indicated. Interface of Domain 1 and β-helix at position 315 and constriction within the helix at position 722 of the primary sequence of EspP are highlighted.

To gain insight into the structural properties responsible for the functional differences between wild-type and mutant EspP proteins, we employed homology modelling of the passenger domain of EspP and used the resulting structure as a template to analyse the structure-function relationships in EspP. Comparative homology modelling is, however, challenging when sequence identity of the template and target is <30% [21], [22]. Furthermore, protein structures have been resolved for only few autotransporters [23]–[26] and only one SPATE protein, Hbp (haemoglobin protease) [27]. Recently, Fernandez-Fuentes et al. described an iterative algorithm to comparatively model target proteins with low sequence identity to the template [28]. This approach enabled us to create an overall consistent model of EspP as evidenced by evaluation of model quality (Table S1, supplementary material). Despite the low (27%) sequence identity between EspP and the chosen template Hbp, the resulting model displays large similarities when compared with the structure of Hbp. The predicted EspP structure harbours an extensive slightly kinked β-helical structure C-terminal from Tyr^315^ of the primary sequence of the holoprotein with 23 right-handed beta-roll turns (Hbp: 24) (Fig. 2). In contrast to Hbp, the β-helix is interrupted at Lys^722^, leading to a constriction at this position. The N-terminal fourth forms a distinct large globular domain which is connected to the C-terminal part of the passenger domain at position 315 and is in close contact with the β-helix via several loop regions. A second globular domain protrudes from the β-helical stem between positions 515 and 562. The corresponding domain in Hbp is 75 amino acids in size [27], slightly larger than in the EspP model and includes, in contrast to EspP, an aromatic binding pocket suggesting different functionality. According to the nomenclature of the corresponding globular domains in the template Hbp, we designated the large and the small globular domain in the EspP model Domains 1 and 2, respectively (Fig. 2).

### Structural analysis of secretion-ablating mutations in EspP passenger domain

#### i) Secretion-deficient mutants in the N-terminal part of the passenger domain

**Figure 3:**
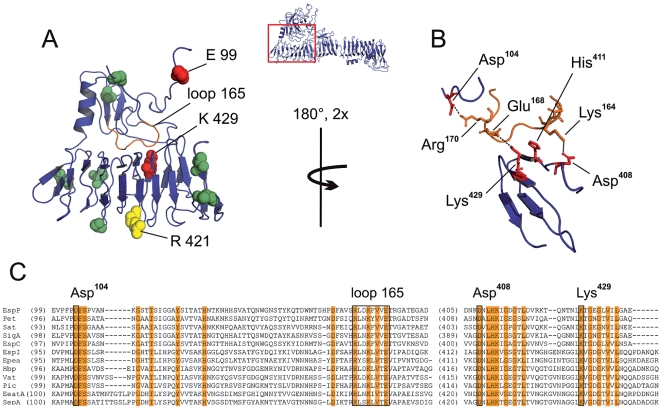
Transport activity of inserts in the N-terminal part of the passenger domain. A. Ribbon diagram of junction between Domain 1 and β-helix. Localization of this region is illustrated in the overview of the passenger domain. Insert positions of linker mutants in the junction are highlighted by coloured spheres, indicating transport activity of the respective constructs (red: transport-inactive, yellow: residual activity, green: activity comparable with wild-type). Constructs displaying loss of function are highlighted by construct names. Loop 165 is marked in orange. B. Detailed representation of interactions leading to stabilisation of the junction region (twofold magnification, view rotated 180°). Salt bridges are indicated by dashed lines, residues involved in stabilising interactions are named and illustrated in stick representation. Loop 165 is given in orange. C. Sequence alignment of residues in SPATE proteins forming the junction region. Overall conserved residues are highlighted in orange. Residues mediating inter-domain interactions as described in B are named and marked by black frames. Key residues involved in interaction are highly conserved in SPATE proteins.

Inserts in transport deficient mutants in the N-terminal half of the passenger domain (E99 and K429) are located in proximity in the EspP model despite their remote insert positions in primary sequence. Insert K429 is predicted to be located in the β-helical stem directly facing globular Domain 1, whereas insertion of construct E99 is within Domain 1 adjacent to a loop, which is closely apposed to the opposite β-helix (Fig. 3A). In accordance with its position in the primary sequence of EspP, we designated this region loop 165. Residues up- and downstream of this loop are predicted to be threaded closely through the neighbouring regions of Domain 1, most probably stabilising the structure. One of the major interactions in the model structure is thereby formed by a salt bridge between Asp^104^ and Arg^170^ upstream of loop 165 (Fig. 3B). Loop 165 itself forms a complex salt bridge with the opposing β-helix, where the residues Lys^164^ and Glu^168^ interact with Asp^408^ and Lys^429^, respectively. His^411^, located in the centre of the salt bridge, is presumed to further stabilise the interaction (Fig. 3B).

To further evaluate these findings and to assess if this structural feature is conserved within the SPATE family, we built a model of the related passenger domain of Pet (plasmid-encoded toxin of enteroaggregative *E. coli*) and compared the structural properties of EspP, Pet and the Hbp crystal structure, as well as sequence conservation of the SPATE proteins within this region. All structures displayed a loop region similar to loop 165 of EspP, forming intimate contact to the neighbouring β-helix via salt bridges (Fig. S1A). Furthermore, key residues presumed to be involved in stabilization of the loop region and formation of salt bridges between loop 165 and β-helix are highly conserved among members of the SPATE family, whereas sequence conservation of the surrounding regions is less pronounced (Fig. 3C). Insert position in the secretion incompetent construct E99 is juxtaposed to the highly conserved sequence DFS at positions 104–106 (Fig. 3C), where Asp^104^ is predicted to form a salt bridge with Arg^170^ stabilizing loop 165. We assume that the linker insertion at this position causes localised misfolding and lack of stabilizing interactions with loop 165 leading to impaired contact between the β-helical stem and the globular Domain 1. The insert in the transport-deficient construct K429 is in a conserved sequence motif directly involved in the formation of the salt bridge with loop 165, whereas the insert position in R421, which shows residual transport activity, is on the contralateral side of the β-helix. Mutants where insertions occurred on either side of this region showed no loss of transport activity (Fig. 3A), suggesting a specific interaction between loop 165 and this part of the β-helical stem.

Taken together, these results suggest that the interaction of the globular Domain 1 with the β-helical stem mediated by loop 165 is an overall conserved structural feature of SPATE proteins essential for the efficient secretion of the passenger domain. These findings are further supported when we display results of a similar mutagenesis approach recently applied to the related autotransporter Pet [29] on our Pet homology model. Both linker inserts leading to the lack of transport activity within the N-terminal half of the passenger domain reported in that study (I114 and A452, respectively) are located in close proximity of the respective transport-deficient constructs of EspP. Similar to EspP, insert I114 is situated in a conserved region presumed to stabilize loop 165, whereas construct A452 occurs in the opposing β-helix region (Fig. S1B).

#### ii) Linker insertions in Domain 2 leading to loss of secretion

**Figure 4:**
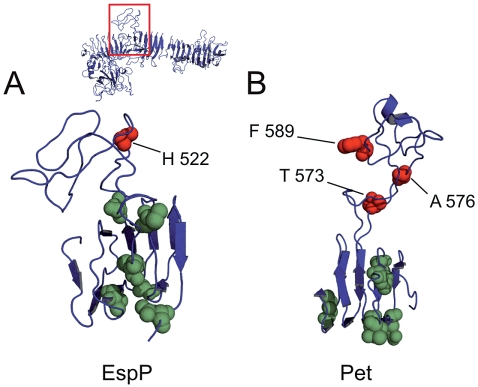
Transport activity of insertions in the central part of the passenger domain. A. Linker inserts surrounding Domain 2 in EspP are depicted as coloured spheres. Transport active constructs are illustrated in green, secretion-incompetent mutants are marked red. Insert H522 in the loop of Domain 2 leads to loss of autotransport activity, whereas neighbouring inserts in the β-helical stem do not influence secretion competence. B. Display of mutagenesis results from Dutta et al. (2003) on our Pet model. Insertions in the respective Domain 2 lead to loss of transport functionality, while insertions in the β-helix core are permissive to transport activity. Localisation of Domain 2 within the passenger domain structure is exemplified for EspP.

Mutant H522 in the central portion of the EspP passenger domain displays loss of autotransport activity (Fig. 1A), whereas mutants with insertions in surrounding regions demonstrated normal secretion efficiency. Notably, the insert in H522 is in the small globular Domain 2 of the EspP model, whereas the neighbouring mutants are located in the adjacent β-helix (Fig. 4A). To confirm these findings and to investigate more broadly if SPATE proteins are susceptible to alterations in Domain 2 concerning autotransport activity, we again overlaid the recent mutagenesis study of Pet [29] on our Pet model. Analysis of the corresponding linker mutants provided results comparable to EspP, with transport inactive Pet mutants T573, A576 and F589 located within Domain 2 of our Pet model, and active mutants in the flanking β-helical core region (Fig. 4B). Alignment of the corresponding sequences encoding Domain 2 displayed, however, a pronounced heterogeneity among the SPATE proteins, suggesting different functions of this part of the passenger domain (data not shown). We noted that the positions of Domain 2 are slightly shifted in the models of EspP and Pet. While in EspP Domain 2 is formed by amino acid 515–562, in Pet residues 571–629 are involved in the formation of this domain. Though a modelling artefact cannot be excluded as an explanation for this difference in view of the low sequence similarity to the template Hbp, our finding of transport-deficient Pet and EspP mutants localised in Domain 2 of both proteins and active mutants in the surrounding regions supports the obtained modelling data. In conclusion, the data suggest that minor alterations in the exposed Domain 2 are critical for the autotransport phenotype of SPATE proteins.

#### iii) Secretion-deficient mutants in the C-terminal part of the passenger domain

**Figure 5:**
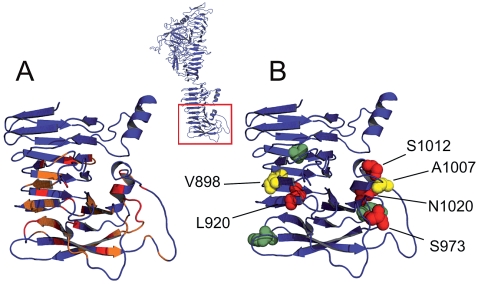
Sequence conservation and transport activity of constructs in the C-terminus of the passenger domain. A. Sequence conservation in linker region of the passenger domain. Residues displaying overall sequence identity are coloured in red, sites showing at least 80% sequence similarity are given in orange. B. Insert positions in transposon mutants highlighted as coloured spheres. Transport-active constructs are illustrated in green, secretion-incompetent mutants are marked in red and constructs displaying residual transport activity are coloured yellow. Insert position is given for transport-inactive constructs.

The C-terminal 100 amino acids of the EspP passenger domain accumulated an abundance of transport-incompetent mutants. This region has been proposed to play a decisive role in autotransport activity, and has been termed linker region [30]–[32]. Alignment of this part of the passenger domain in our model illustrated an increase in sequence conservation among proteins of the SPATE family compared to the N-terminal part, further indicating that this region might encode a shared functional aspect. Display of conserved residues on the EspP model demonstrates, however, that sequence conservation was not distributed equally in the C-terminal part of the EspP passenger, but accumulated mostly in stacked residues within flanks of the β-helix and the C-terminal end of the passenger domain (Fig. 5A). Comparison of insert positions and sequence conservation within the C-terminal linker region demonstrated that mutants harbouring inserts at positions of overall conservation showed loss of transport activity, whereas insertion into heterogeneous sequence parts was, in general, compatible with autotransport activity (Fig. 5B).

We therefore determined if conservation of key residues correlates with surface properties of the SPATE proteins, but there was no consistent motif when comparing hydrophobicity and/or charge of the respective linker regions of EspP, Pet and Hbp (data not shown). We conclude therefore that conservation of the overall structure, rather than specific surface properties, is crucial to preserve autotransport activity of the linker region.

Interestingly, we noted that several transport-deficient transposon mutants harboured inserts near a unique point mutation present in the *espP*β gene of the recently characterized strain 89/04 [16]. Though the corresponding EspP protein of this strain differs only in the point mutation R1005Q from other members of the EspPβ subtype, we were not able to detect, in contrast to other samples from this subtype, EspP protein in the supernatant of this strain. However, a R1005Q site-directed mutant in pB9-5 did not diminish transport activity, indicating that other factors might be responsible for the lack of extracellular EspP in strain 89/04. Indeed, *espP*β in strain 89/04 is not transcribed as evidenced using reverse transcription (RT)-PCR, suggesting that the prime defect in non-secretion of EspP is absence of gene expression, rather than altered protein sequence.

### Structural analysis of transport-deficient mutants in β-domain

**Figure 6:**
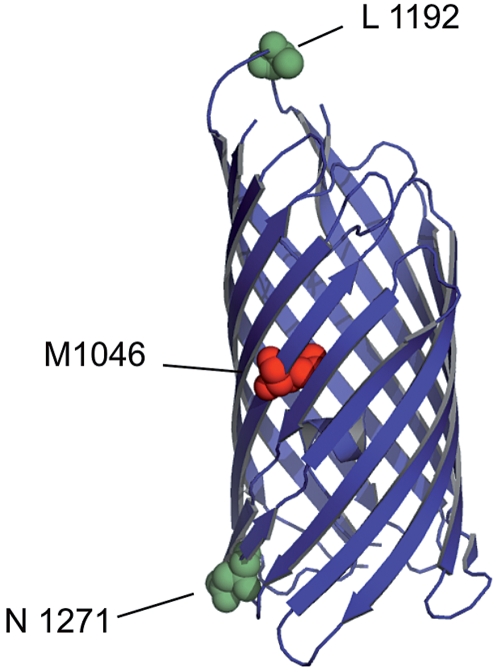
Transport activity of linker inserts in the β-domain. Three linker mutants locate inserts in the β-barrel of EspP as indicated by coloured spheres. Inserts within loop regions were permissive to transport (illustrated green), while insertion in the central part of the barrel lead to loss of transport activity (indicated red).

Using linker scanning mutagenesis, we obtained three mutants (M1046, L1192 and N1271) in the β-domain, the C-terminal part of the EspP holoprotein involved in transport through the outer bacterial membrane. Using the recently solved structure of this domain [14], we demonstrated that the transport-deficient mutant M1046 harbours the linker insert in the first β-sheet, whereas both constructs displaying normal transport activity (L1192 and N1271) show linker insertions within loop regions (Fig. 6). These data underline the crucial role of a correctly folded β-barrel for autotransport of EspP, whereas loop regions within the β-domain are more tolerant to structural alterations.

### Structural properties of proteolytic activity of theEspP passenger

**Figure 7:**
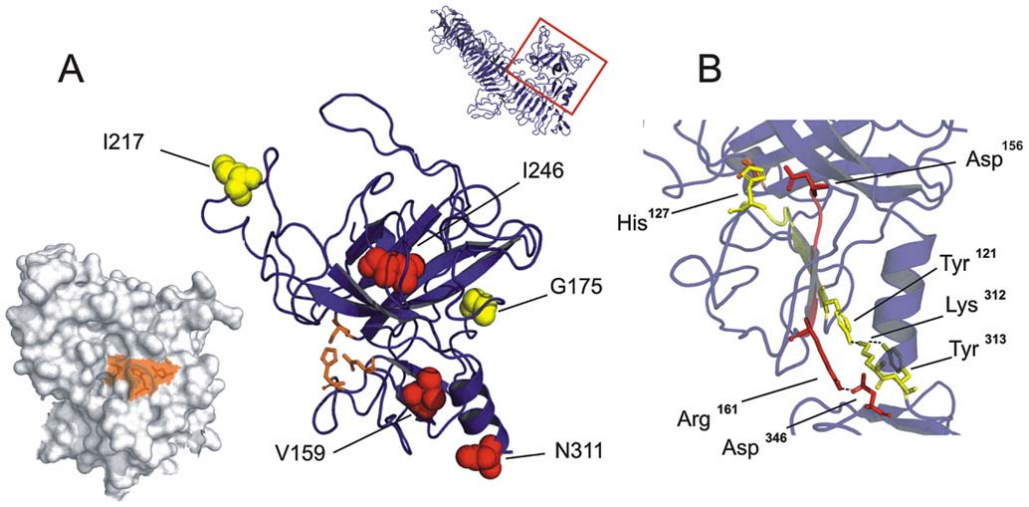
Analysis of proteolytic activity in Domain 1. A. Left: Display of the catalytic triad on the surface of Domain 1. Residues forming the active centre are given as sticks and coloured orange. All residues of the catalytic triad are surface exposed. Right: Insert positions of linker in Domain 1 are marked by coloured spheres indicating the proteolytic activity of the respective constructs. Red spheres symbolise complete loss of activity in the corresponding construct; yellow spheres indicate highly reduced residual activity. Residues forming the catalytic triad are shown as orange sticks. B. Interaction between Domain 1 and the interfacing α-helix. Residues involved in stabilisation of His^127^ (Tyr^121^, Lys^312^ and Tyr^313^) are shown as yellow sticks, amino acids mediating stabilisation of Asp^156^ (Arg^161^ and Asp^346^) are given in red.

To verify our finding that His^127^, Asp^156^ and Ser^263^ form the catalytic triad of EspP, we displayed the positions of the three amino acids on the homology model of EspP. This demonstrated that all these residues are located in close proximity on the surface of Domain 1, underlining their role in the formation of the active centre of EspP (Fig. 7A). Transposon mutants were proteolytically inactive when inserts were located near the catalytic triad. Mutants G175 and I217, which showed residual proteolytic activity, harboured the linker insert further from the active centre in the model structure, supporting our observation that the loss of proteolytic activity is only observed when structural alterations occur close to the active centre (Fig. 7A). To our surprise, the proteolytically inactive transposon mutant N311, and the site-directed double mutant Y313F/N316K, mimicking the alterations presumably responsible for the lack of proteolytic activity in EspPβ and EspPγ [16], are located in the region interfacing Domain 1 and the β-helical core directly downstream the linking α-helix of the model structure. In addition, we noted that Arg^161^ upstream Asp^156^ and Tyr^121^ downstream His^127^ are predicted to contact with residues in this interfacing region, suggesting that these interactions might contribute to functional positioning of residues Asp^156^ and His^127^ as parts of the active centre (Fig. 7B). Arg^161^ is conserved in all SPATE passenger domains (see also Fig. 3C, sequence conservation of loop 165) and is predicted to build a salt bridge with Asp^346^, whereas Tyr^121^ interacts via hydrogen bonds with Lys^312^ and Tyr^313^ (Fig. 7B). These results suggest that His^127^ and Asp^156^, involved in the formation of the catalytic triad of EspP, are stabilised by interactions with the interfacing region between Domain 1 and the β-helix. Decreased proteolytic activity of mutants N311 and Y313/N316K might therefore be caused by improper formation of the catalytic triad as a result of reduced stabilizing hydrogen bonds between Tyr^121^, Lys^312^ and Tyr^313^.

## Discussion

We used a combination of mutagenesis techniques and homology modelling to gain insight into mechanisms underlying functional variations in the previously identified EspP subtypes [16]. This approach allowed the identification of critical modifications in the EspP subtypes β and δ leading to lack of proteolytic activity. Both mutations, Y313F and N316K, have been observed in all characterised samples of the above subtypes, whereas none of the proteolytically active subtypes, EspPα or EspPγ, harboured these modifications [16]. It is, however, noteworthy that the corresponding double mutant Y313F/N316K only incompletely reproduces the phenotype observed in EspPβ and EspPδ. EspP proteins of the respective subtypes produced by STEC strains displayed complete lack of proteolytic activity [16], whereas mutant Y313F/N316K demonstrated reduction of activity of approximately 50%. It is therefore likely that further modifications might contribute, though indirectly, to the observed phenotype in STEC strains. In contrast, lack of secretion of EspPβ from the prototype strain 89/04 [16] does not result from modifications on protein level, but is caused by impaired gene expression. This finding might provide a fundament for the analysis of regulatory elements of EspP in future studies.

Concerning the effect of linker insertions on secretion efficiency of EspP in general, we observed that mutations in three different and definable regions of the folded passenger ablated the secretion phenotype. First, mutants showing linker insertions within a limited junction region between Domain 1 and the nearby β-helix were no longer able to perform autotransport, whereas inserts juxtaposed to this region did not affect this function. These results suggest that formation of a compact tertiary structure, mediated by different noncovalent interactions between loop 165 in Domain 1 and β-helix, contributes to the efficient secretion of the passenger domain to the extracellular compartment. Interestingly, paired cysteine mutants in Hbp covalently connecting Domain 1 and β-helix by newly introduced disulphide bridges in the junction region eliminate autotransport activity [33]. Consequently, it has been assumed that either tolerance towards folding is limited or flexibility of a folded passenger domain plays an important role for secretion. Based on our findings, we favour the hypothesis that the passenger domain folds at least at one step during secretion and that stabilizing interactions between the folded Domain 1 and β-helix are indispensable. Furthermore, the obtained tertiary structure obviously needs to maintain a certain degree of flexibility as provided by the relatively weak interactions mediated via salt bridges. In contrast to that, covalent binding of the folded domains, as mediated by proximal cysteine mutants, are incompatible with autotransport.

Our hypothesis that folding is critical for the secretion of the passenger domain was further supported by the observation that insertions within Domain 2 also lead to the loss of transport function, whereas modifications in the surrounding β-helical stem did not. The finding that minor alterations of the small Domain 2 (introduction of 5 residues in a domain of ∼50 amino acids) influence secretion was unexpected as we assume that the overall modification of the tertiary structure is minimal. While loss of interactions between Domain 1 and β-helix are presumed to substantially change folding of the passenger domain, the effects of linker inserts in the protruding Domain 2 are most probably only local. A direct involvement of Domain 2 in autotransport is, however, rather unlikely, because this part shows only low sequence similarity among SPATE proteins, suggesting that no shared functional feature is encoded in this part of the protein. Moreover, deletion mutants lacking Domain 2 have been reported to be secreted efficiently [32]–[34]. Our findings were confirmed for Pet when we displayed the corresponding linker mutants reported by Dutta and colleagues [29] on our Pet model. Linker insertions in Domain 2 of Pet also prevented secretion, suggesting that a (potentially conserved) functional aspect in the autotransport mechanism of SPATE proteins is susceptible to alterations of exposed regions like Domain 2.

In this context, the recently discovered Omp85/YaeT complex, essential for the integration of β-barrel proteins (including autotransporters) in the outer membrane [35], [36], might play a yet unknown role in the autotransport secretion process. A recently proposed model hypothesized that Omp85/YaeT is not only involved in the formation of the integration-competent folded state of autotransporter β-domains, but furthermore contributes to the translocation of folded passenger domains through the outer membrane [37]. This model is in accordance with the observation that the pore size (∼2.5 nm) of the Omp85/YaeT complex is large enough to allow the translocation of at least partially folded proteins [38].

The C-terminal linker region of passenger domains has been proposed to be fundamental for autotransport activity of a variety of proteins [30]–[32], [39], [40]. Conserved residues, localization of autotransport-deficient mutants, and analysis of surface properties in our study provide data that conservation of structural elements, rather than surface characteristics per se, is crucial for autotransport functionality in this region. A recent study suggested that hydrophobic residues of the EspP linker domain are essential for the outer membrane transport [32]. Display of secretion incompetent mutants obtained in that study on our EspP model demonstrates, however, that mutations mostly occurred in β-sheets of the EspP linker domain. Moreover, most alterations in transport-incompetent mutants changed hydrophobic residues to proline, which is known to be deleterious for the formation of β-sheets [41], [42]. We propose therefore that inhibition of formation of β-sheets rather than changed hydrophobicity causes loss of autotransport activity in the linker region.

Notably, the C-terminal end of the EspP passenger resembles to a certain extend the POTRA (polypeptide-transport-associated) domains of the TpsB protein FhaC, the transport cofactor in the two-partner secretion system of FHA (filamentous hemagglutinin) [43]. Though the α-helical part typical of POTRA domains is missing, the residual structure is similar with key residues conserved among SPATEs forming the structural backbone. We therefore hypothesize that the linker part of EspP might be involved as a periplasmic autochaperone in folding or maturation during secretion similar to the POTRA domain in the TpsB proteins, which is, strikingly, also situated directly N-terminal to the part forming the β-barrel.

In summary, our data demonstrate that constructs lacking proteolytic activity are confined to the N-terminal third of the EspP passenger domain. All proteolytically inactive mutations were limited to the globular Domain 1, providing evidence that this part of the passenger domain solely encodes the proteolytic activity. The experimental confirmation of His^127^, Asp^156^ and Ser^263^ as residues forming the proteolytically active site in EspP further supports the hypothesis that Domain 1 comprises a classical serine protease with a catalytic triad as structural basis for proteolytic activity. Employing the DALI server [44] to search for structural homologues of Domain 1 revealed that residues 102–313 are quite similar to porcine pancreatic elastase (PDB: 9est), with a Z-score of 17.0 (scores >2.0 are considered significant), suggesting that the proteolytic domain in the EspP passenger might be elastase-like. Future studies will address, if EspP not only shows structural similarities with elastases but also shares functional aspects with this serine protease family.

## Materials and Methods

### Bacterial strains and plasmid constructs

*E. coli* K12 strain DH5α was host for all constructs used in this study. Functional active EspP was obtained from clone DH5α/pB9-5, which contains *espP* from STEC O157:H7 strain EDL933 [7].

### Linker permissive transposon mutagenesis

Plasmid pB9-5 was subjected to randomized mutagenesis *in vitro* with the Tn7-derived GPS-LS transposon using the GPS-LS linker scanning system (New England Biolabs, Beverly, USA) according to the manufacturer's instructions. Briefly, purified plasmid pGPS-4 was used as donor plasmid for the transposition reaction to insert a chloramphenicol resistance cassette (Camp^r^) into pB9-5. The mutagenized plasmids were transformed into *E. coli* DH5α that was incubated on Luria Bertani (LB) agar plates containing kanamycin and chloramphenicol. Colonies grown on selective media were selected for screening for insertions in the open reading frame of *espP* and estimation of insert position using PCR with one fixed forward primer (5′-cgc tgt ttc tga att atc cgg ca-3′) annealing in the 3′-end of the *espP* gene and two primers annealing in the borders of the insertion element, respectively (Tn-R, 5′-act tta ttg tca tag ttt aga tct att ttg-3′ and Tn-L, 5′-ata atc ctt aaa aac tcc att tcc acc cct-3′). Plasmids from constructs with (Camp^r^) insert in the *espP* gene were purified using Qiagen Spin Miniprep Kit (Qiagen, Hilden, Germany). Plasmid DNA was subsequently digested with *Pme*I to remove the chloramphenicol resistance gene and plasmid fragments were separated by agarose gel electrophoresis, purified using the Prep-A-Gene Kit (Bio-Rad, Hercules, USA), religated and transformed into *E. coli* DH5α. Sequence analysis was conducted to determine the position of the remaining 15 bp linker using primer Tn-R and Tn- L. Based on sequence data, all constructs have been analysed for in-frame insertion of the linker resulting in a five amino acids prolonged EspP protein.

### Site-directed mutagenesis

Site-directed mutagenesis was performed either using the Quick change II XL kit (Agilent-Stratagene, Waldbronn, Germany) or Phusion kit (New England Biolabs, Beverly, USA). Briefly, purified plasmid pB9-5 was used as template for the Quick change II XL protocol and amplified in a linear amplification reaction using the mutagenic primers (H127A: f: 5′-cag tat cac agc cac agc caa tac gaa aaa cca cca ctc a-3′ and r: 5′-gtc ata gtg tcg gtg tcg gtt atg ctt ttt ggt ggt gag t-3′; D156A: f: 5′-gaa tac ttc aca tcc tgc ttt tgc agt atc ccg act tga c-3′ and r: 5′-ctt atg aag tgt agg acg aaa acg tca tag ggc tga act g-3′; S263A: f: 5′-cat tgc ctc tca ggg tga cgc cgg ttc agc act gtt cg-3′ and r: 5′-cga aca gtg ctg aac cgg cgt cac cct gag agg caa tg-3′). Template DNA was digested with *Dpn*I and the newly synthesized plasmid was transformed into *E. coli* DH5α using electroporation. The Phusion kit was applied for constructs Y313F/N316K, and R1005Q using mutagenic primers (Y313F/N316K: Y313F f: 5′-tgg ata tgt cag ggg ctc agg -3′, and r: 5′-cgt tgt aag aaa act tgt tct tca gg-3′; N316K f: 5′-att ctt aca aag tgg ata tgt cag gg-3′, and r: 5′-act tgt tct tca ggt tgt caa tgg-3′; R1005Q f: 5′-cgc cct gtt ctc tgt tga cta taa ag-3′, and r: 5′-gcg gca ttc tgg gtt gct tcc t-3′). Again, purified plasmid pB9-5 was used as template for the amplification reaction. PCR product was ligated using Quick T4 DNA ligase and transformed into *E. coli* DH5α using electroporation according to recommendations in Phusion manual. For each construct three individual colonies were subjected to plasmid purification and sequence analysis ensuring successful mutagenesis.

### Purification of EspP from culture supernatants and whole-cell extracts

For the purification from culture supernatants strains were grown overnight in 100 ml of LB broth at 37°C with vigorous shaking. The cultures were centrifuged (6,000×g, 30 min, 4°C) and supernatants were passed through 0.20 µm pore-size filters (Corning, Corning NY, USA). Proteins were precipitated (1 h, 4°C) by adding ammonium sulphate (Merck, Darmstadt, Germany) to 55% saturation. The precipitate was collected by centrifugation (6,000×g, 30 min, 4°C) and pellet was dissolved in 500 µl of 10 mM HEPES (*N*-2-hydroxyethylpiperazine-*N*'-2-ethanesulfonic acid) buffer containing 150 mM NaCl (pH 7.4). EspP was purified using HiTrap Benzamidine FF columns (GE Healthcare, Freiburg, Germany) according to the manufacturer's instructions. The fractions enriched for EspP were collected and concentrated using 10 kDa Vivaspin spin-down filter (Vivascience-Sartorius, Göttingen, Germany). For the analysis of EspP in whole-cell extracts strains were grown in 25 ml LB broth to an OD_600_ of 1.5, centrifuged (4,000×g, 20 min, 4°C), washed with phosphate buffered saline and resuspended in 4× Laemmli sample buffer. Cells were sonicated three times for 1 min and boiled for 10 min at 99°C.

### SDS-PAGE and immunoblotting

Detection of EspP in crude culture supernatants and purified EspP samples was performed as described previously [16]. Briefly, proteins were separated by SDS-PAGE, transferred to nitrocellulose membrane (Schleicher & Schuell, Dassel, Germany) and detected with rabbit anti-EspP antibody (diluted to 1∶1.000) [7] and a horseradish peroxidase-conjugated goat anti-rabbit antibody (diluted to 1∶40.000) (Dianova, Hamburg, Germany). Bound antibodies were visualised by incubation with SuperSignal West Pico chemiluminescent substrate (Pierce, Rockford, USA), followed by the detection of the chemiluminescence signal using ChemiDoc XRS imaging system (Bio-Rad, München, Germany). The detection limit of the assay was ≥50 ng of protein. Detection of EspP was performed in crude culture supernatants and the purified samples, respectively, to rule out the possibility that EspP was lost during purification. EspP from each construct was purified and analysed in triplicate.

### Analysis of proteolytic activity

One microgram of purified EspP was incubated (15 h at 37°C) with a 2 mM solution of the para-nitroaniline-conjugated oligopeptide substrate alanine-alanine-proline-leucine (Ala-Ala-Pro-Leu-pNA) (Bachem, Weil am Rhein, Germany) in 10 mM HEPES buffer with 150 mM NaCl, pH 7.4. Reactions were performed in 96-well microtiter plates in total volumes of 100 µl. The absorbance was determined at 0 min and 15 h at 405 nm using a microplate reader (Dynex Technologies, Chantilly, USA). Samples were defined as proteolytically active when the difference of the absorbance readings was ≥0.03 units. The EspP-producing clone DH5α/pB9-5 and the vector control strain DH5α (pK18) without the *espP* insert were used as positive and negative control, respectively. Three individual purifications of each construct were analysed. For the analysis of proteolytic activity against porcine pepsin A one microgram EspP was incubated with 25 µg pepsin A for 15 h at 37°C. Substrate cleavage was analysed using SDS-PAGE followed by Coomassie staining.

### Homology modelling

Comparative homology modelling of the EspP passenger domain was performed employing the M4T server [28]. The modelling approach is based on an iterative algorithm for automated target-to-template alignment followed by model building using the MODELLER program [45] integrated in the M4T package and subsequent structural optimization of the obtained model. The sequence of the passenger domain of EspP from STEC O157:H7 strain EDL933 [7] was used as target for model building. The structure of Hbp (PDB: 1wxr) [27] was chosen by M4T as template for modelling. Homology modelling of the Pet passenger domain was performed accordingly, using the sequence published by Eslava and coworkers [46]. Evaluation of the obtained models was performed using ANOLEA [47], Prosa2003 [48], PROCHECK [49] and QMEAN [50]. Figures were prepared using Pymol (DeLano Scientific).

### Two-step RT-PCR

STEC strains EDL933 (O157:H7) and 89/04 (O7:H18) were inoculated into 30 ml of LB broth and grown at 37°C with vigorous shaking (180 rpm) for approximately 150 minutes. Bacteria were collected by brief centrifugation (20,000×g, 20°C) and RNA was extracted using High Pure RNA Isolation kit (Roche, Mannheim, Germany) according to the manufacturer's instructions. Two µg of bacterial RNA were treated with 1 µg of DNAse I (Stratagene, La Jolla, USA) for 15 min at room temperature. Reverse transcription was performed by adding 1 µl of Superscript II (Invitrogen, Karlsruhe, Germany) and incubation at 42°C for 50 minutes followed by incubation at 70°C for 15 minutes. The reaction was stopped by chilling on ice. Subsequently, 1 µl aliquots of the resulting cDNA were amplified using primers EspP fwd (5′-gct cca ccc tga aac tac cg-3′) and EspP rev (5′-cgt tca agt gcc tgc tgt tt-3′). *gap*A was used as a positive control and was amplified using primers GapA fwd (5′-gtt gtc gct gaa gca act gg-3′) and GapA rev (5′-agc gtt gga aac gat gtc ct-3′). In negative controls, RNA was omitted from the cDNA synthesis before PCR amplification. PCR products were separated in 2.0% agarose gel and visualised by staining with ethidiumbromide.

## Supporting Information

Figure S1 Interaction between loop 165 and β-helix is conserved in SPATE proteins. A. Representation of interfacing junction region in the SPATEs EspP, Hbp and Pet. Salt bridges are marked with dashed lines, residues involved in formation of stabilizing interactions are shown as sticks, and respective residues are labelled. Key residues involved in interactions are conserved on sequence level as indicated in Fig. 2 and display large structural similarities. The respective loops 165 are marked in orange. B. Linker insertion in junction region of Pet interferes with transport activity. Inserts at positions preventing the correct formation of junction region lead to loss of transport activity in Pet, as evidenced by analysis in the homology model. Position of inserts in the respective constructs is illustrated as red spheres (constructs I114 and A452), surrounding linker mutants permissive to secretion are illustrated in green. Data of the linker mutagenesis study of Pet reported previously, (Dutta et al., 2003) have been displayed on the Pet homology model.
(10.04 MB TIF)

Table S1 Evaluation of model quality. Evaluation of model quality of EspP and Pet using Procheck and QMEAN. Both programs confirm that valid models of EspP and Pet were obtained with quality parameters comparable to the template structure (PDB: 1wxr) derived from Hbp. Procheck parameters display 82–86% of the residues in the most favoured region of the Ramachandran Plot and less than 1% in disallowed regions with only marginal differences between template and model scores. The QMEAN program was used for comparison of model quality of the obtained homology models. Scores of the chosen models of EspP and Pet are comparable to the score for the template structure Hbp.
(0.03 MB DOC)
