# Supplementary figures and images for: Structure and Function Relationship of the Autotransport and Proteolytic Activity of EspP from Shiga Toxin-Producing Escherichia coli
Source: PLoS One. 2009 Jul 1;4(7):e6100. doi: 10.1371/journal.pone.0006100 (PMC2700255; doi:10.1371/journal.pone.0006100)

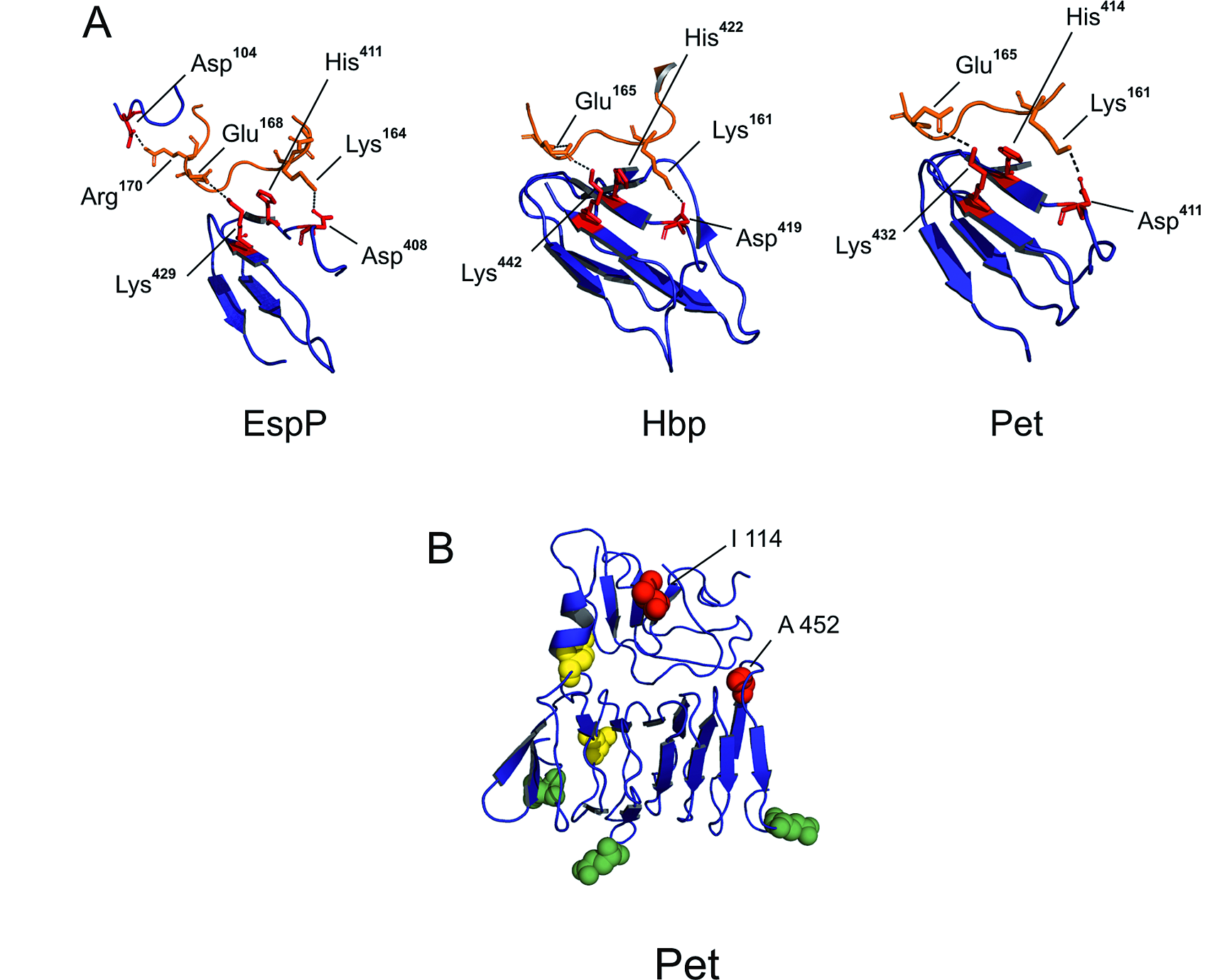

Supplement: Figure S1 — Interaction between loop 165 and β-helix is conserved in SPATE proteins. A. Representation of interfacing junction region in the SPATEs EspP, Hbp and Pet. Salt bridges are marked with dashed lines, residues involved in formation of stabilizing interactions are shown as sticks, and respective residues are labelled. Key residues involved in interactions are conserved on sequence level as indicated in Fig. 2 and display large structural similarities. The respective loops 165 are marked in orange. B. Linker insertion in junction region of Pet interferes with transport activity. Inserts at positions preventing the correct formation of junction region lead to loss of transport activity in Pet, as evidenced by analysis in the homology model. Position of inserts in the respective constructs is illustrated as red spheres (constructs I114 and A452), surrounding linker mutants permissive to secretion are illustrated in green. Data of the linker mutagenesis study of Pet reported previously, (Dutta et al., 2003) have been displayed on the Pet homology model. (10.04 MB TIF) [file pone.0006100.s001.tif]
