# Supplementary material for: Structure and Function Relationship of the Autotransport and Proteolytic Activity of EspP from Shiga Toxin-Producing Escherichia coli
Source: PLoS One. 2009 Jul 1;4(7):e6100. doi: 10.1371/journal.pone.0006100 (PMC2700255; doi:10.1371/journal.pone.0006100)
Supplement: Table S1 — Evaluation of model quality. Evaluation of model quality of EspP and Pet using Procheck and QMEAN. Both programs confirm that valid models of EspP and Pet were obtained with quality parameters comparable to the template structure (PDB: 1wxr) derived from Hbp. Procheck parameters display 82–86% of the residues in the most favoured region of the Ramachandran Plot and less than 1% in disallowed regions with only marginal differences between template and model scores. The QMEAN program was used for comparison of model quality of the obtained homology models. Scores of the chosen models of EspP and Pet are comparable to the score for the template structure Hbp. (0.03 MB DOC) [file pone.0006100.s002.doc]

**Table S1**

|  | **Procheck** (Ramachandran Plot) | | | | **QMEAN** |
| --- | --- | --- | --- | --- | --- |
| Protein structure | Core  (%) | Allowed  (%) | Generous  (%) | Disallowed (%) | Score |
|  |  |  |  |  |  |
| Hbp (PDB: 1wxr) | 86.1 | 12.9 | 0.7 | 0.3 | -112.8 |
|  |  |  |  |  |  |
| EspP (model M4T) | 82.3 | 13.6 | 3.2 | 0.9 | -84.3 |
|  |  |  |  |  |  |
| Pet (model M4T) | 83.2 | 12.0 | 4.1 | 0.7 | -78.8 |
|  |  |  |  |  |  |

**Table S1.** **Evaluation of model quality of EspP and Pet using Procheck and QMEAN.** Both programs confirm that valid models of EspP and Pet were obtained with quality parameters comparable to the template structure (PDB: 1wxr) derived from Hbp. Procheck parameters display 82-86% of the residues in the most favoured region of the Ramachandran Plot and less than 1% in disallowed regions with only marginal differences between template and model scores. The QMEAN program was used for comparison of model quality of the obtained homology models. Scores of the chosen models of EspP and Pet are comparable to the score for the template structure Hbp.
